# Dysfunctional bronchoalveolar effector memory CD8^+^ T cells in tuberculosis-exposed people living with antiretroviral-naïve HIV infection

**DOI:** 10.1016/j.isci.2024.111137

**Published:** 2024-10-16

**Authors:** Maphe Mthembu, Helgard Claassen, Sharon Khuzwayo, Valentin Voillet, Anneta Naidoo, Jule S. Spillner, Kennedy Nyamande, Dilshaad Fakey Khan, Priya Maharaj, Mohammed Mitha, Zoey Mhlane, Farina Karim, Erica Andersen-Nissen, Thumbi Ndung’u, Gabriele Pollara, Emily B. Wong

**Affiliations:** 1 Africa Health Research Institute, Durban, South Africa; 2 University of KwaZulu-Natal, Medical School, Durban, South Africa; 3 Cape Town HVTN Immunology Laboratory, Cape Town, South Africa; 4 Division of Infection and Immunity, University London College, London, UK; 5 Department of Pulmonology, Inkosi Albert Luthuli Central Hospital, Durban, South Africa; 6 HIV Pathogenesis Programme, The Doris Duke Medical Research Institute, University of KwaZulu-Natal, Durban, South Africa; 7 Ragon Institute of MGH, MIT and Harvard, Cambridge, MA, USA; 8 Department of Medicine, The University of Alabama at Birmingham, Birmingham, AL, USA

**Keywords:** Immunology, Virology

## Abstract

HIV causes susceptibility to respiratory pathogens, including tuberculosis (TB), but the underlying immunological mechanisms remain incompletely understood. We obtained whole blood and bronchoalveolar lavage (BAL) from TB-exposed people in the presence or absence of antiretroviral-naïve HIV co-infection. Bulk transcriptional profiling demonstrated compartment-specific enrichment of immunological processes. Systems-level deconvolution of whole blood from people living with HIV identified elevated type I and type II interferon cytokine activity and T cell proliferation. Transcriptional modules derived from both peripheral blood and sorted BAL immune cells demonstrated an increased frequency of effector memory CD8 T cells in whole BAL samples. Both compartments displayed reduced induction of CD8 T-cell-derived interleukin-17A (IL-17A) in people with HIV, associated with elevated T cell regulatory molecule expression. The data suggest that dysfunctional CD8 T cell responses in uncontrolled HIV may contribute to compromised respiratory immunity to pathogens, a process that could be modulated by host-directed therapies that target CD8 T cell effector functions.

## Highlights

• Paired bulk transcriptional profiling of bronchoalveolar lavage and blood
• In blood, elevated type I/II interferon activity and cell proliferation in HIV
• In bronchoalveolar lavage, enrichment of dysfunctional effector memory CD8^+^ T cells in HIV
• In both compartments, production of IL-17A by CD8^+^ T cells is reduced with HIV

## Graphical abstract

**Figure:**
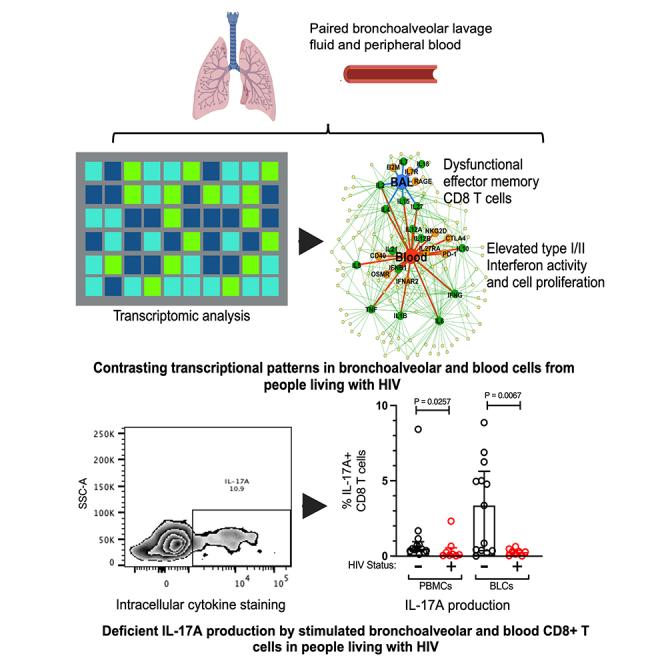

## Introduction

People living with HIV (PLWH) have >18-fold increased risk of respiratory infections, including tuberculosis (TB).^1^ Uncontrolled HIV infection results in several immune perturbations, including CD4 T cell depletion, cytotoxic CD8 T cell expansion,^2^ and elevated cytokine activity^3^ (e.g., type I interferons^4^). Improved mechanistic understanding of HIV’s impact at the site of host-pathogen interactions may offer insights into immune modulation caused by uncontrolled HIV infection, and in turn, identify putative therapeutic targets in PLWH. Most studies have focused on immune perturbations in blood but less on target organs for co-infections, such as the lungs.^5^^,^^6^^,^^7^^,^^8^ Whole compartment transcriptional profiling permits unbiased assessments of disease-mediated perturbation by HIV at the molecular level, while still retaining the ability to deconvolute differences in immune cell frequency, cytokine activity, and other functions.^9^^,^^10^

In this study, we aimed to explore the lung mucosal immune landscape, through transcriptomic profiling of bronchoalveolar lavage (BAL) cells in individuals with untreated HIV infection. We previously established the feasibility of this technique in a small number of individuals undergoing bronchoscopy for clinical investigations.^11^ In the current study, we apply this approach to a larger number of individuals with evidence of *Mycobacterium tuberculosis* (*Mtb*) infection (“latent TB”) in a high TB transmission setting who were otherwise healthy and volunteered for participation in a research bronchoscopy study. We tested the hypothesis that HIV results in compartment-specific immune perturbations that may underlie the susceptibility to TB disease and other respiratory infections.

## Results

### Research bronchoscopy cohort characteristics

Samples from 20 participants (10 HIV-negative and 10 HIV-positive; Table 1) were selected from a larger research bronchoscopy cohort and used to generate whole compartment transcriptomic data. All participants of the research bronchoscopy cohort had no evidence of any active lung disease, prior TB, current or prior tobacco consumption, or other chronic diseases and contributed samples of paired blood and BAL samples (Table S1). Of the 20 participants whose transcriptomic data were analyzed, most were female (60%), and the median age was 30 years (IQR 26–34). Sex and age distribution were comparable between the two groups. All PLWH were naive to antiretroviral therapy at the time of bronchoscopy; median CD4 count in this group was 461 cells/mm^3^ (IQR 256–586) and median VL 29,256 copies/mL (IQR 14,510–33,565; Table 1). Based on sample and data availability, some non-transcriptomic analyses were performed on all samples available from the larger cohort (Table S1).

**Table 1:** Demographic and clinical characteristics of the enrolled participants with whole transcriptomics data

Characteristics	HIV-Negative	HIV-Positive	*p* value^a^
N	10	10	–
Females n (%)	7 (70%)	5 (50%)	0.6499
Age median (IQR)	31 (26–32)	30 (26–35)	0.9872
CD4 counts (cells/mm^3^) median (IQR)	956 (854–1307)	461 (256–582)	0.0003
Viral load (copies/mL) median (IQR)	ND	29256 (14510–33565)	–

a Mann-Whitney between HIV-negative and HIV-positive people; ND, not detected; –, not applicable.

### HIV infection is associated with distinct transcriptional effects in lung and blood

**Figure 1:**
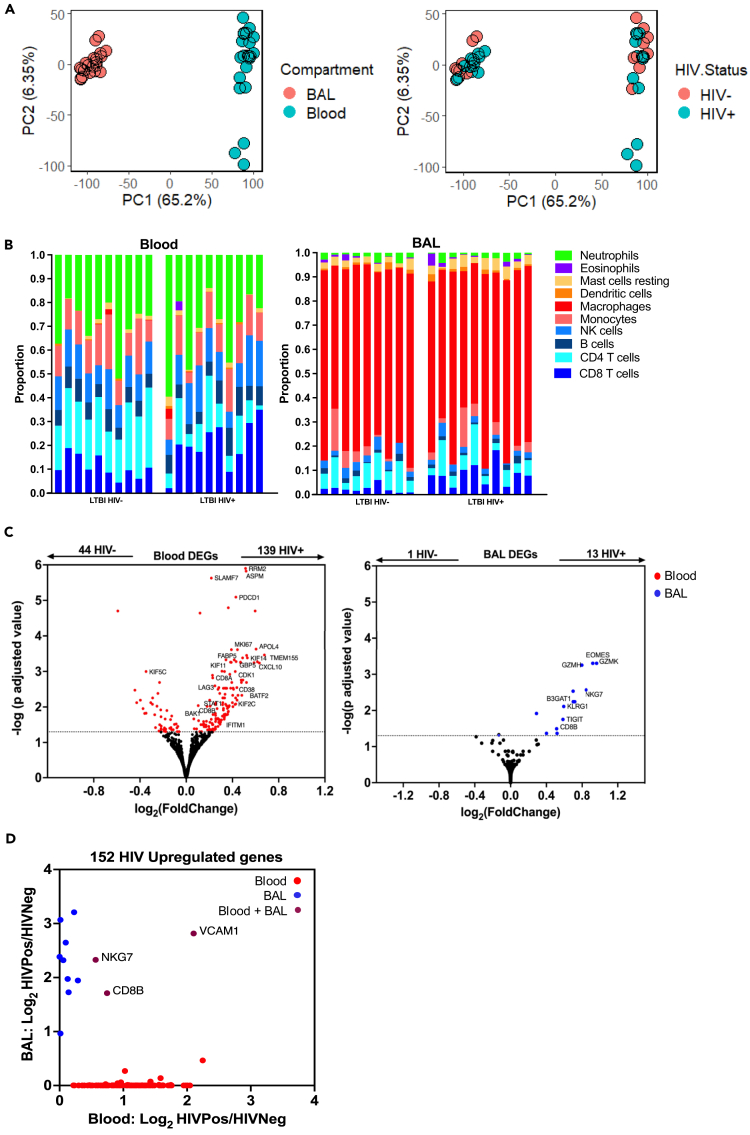
HIV infection associated with different transcriptional profiles in blood compared to the bronchoalveolar space (A) Principal-component analyses of whole-genome level bulk transcriptomes, displaying PC1 and PC2, stratified on the left by compartment (blood or bronchoalveolar [BAL]) and on the right by the presence or absence of HIV infection (*n* = 20, HIV-negative [10] and HIV-positive [10]). (B) Relative proportions of major immune cell types in blood (left) and BAL (right) compartments per participant (columns), stratified by HIV status. (C) Whole-genome differential gene expression transcriptional analysis from blood and BAL samples; volcano plots indicate genes with significantly elevated expression (*p* adjusted <0.05) in HIV infection in blood (red) and BAL (blue) compartments. (D) Dot plot of genes with elevated expression in blood and BAL compartments of people with HIV. Colors represent genes showing elevated expression only in blood (red), only in BAL (blue), or in both compartments (magenta).

Principal-component analyses of baseline blood and BAL transcriptomes from 10 HIV-positive and 10 HIV-negative individuals revealed clear divergence between the two compartments (Figure 1A, left). This difference most likely reflected differential enrichment of immune cell types between the compartments, with neutrophils and lymphocytes predominating in blood and macrophages predominating in BAL as was evident through computational deconvolution of the transcriptomic data (Figure 1B) and cytological interpretation of cellular morphology (Figure S1). We observed greater variance in gene expression within the blood compartment than in BAL, but the effect of HIV in either setting could not be discerned using this approach alone (Figure 1A, right). Therefore, we performed unsupervised whole-genome differential gene expression analyses, identifying 139 upregulated and 44 downregulated genes in blood of PLWH (Figure 1C, left; Table S2), in contrast to 13 upregulated and 1 downregulated, in BAL (Figure 1C, right; Table S3). Of the 152 genes elevated in PLWH across both compartments, only three (2%) were increased in both blood and BAL (Figure 1D), highlighting that HIV infection induced distinct transcriptional changes in the two compartments.

**Figure 2:**
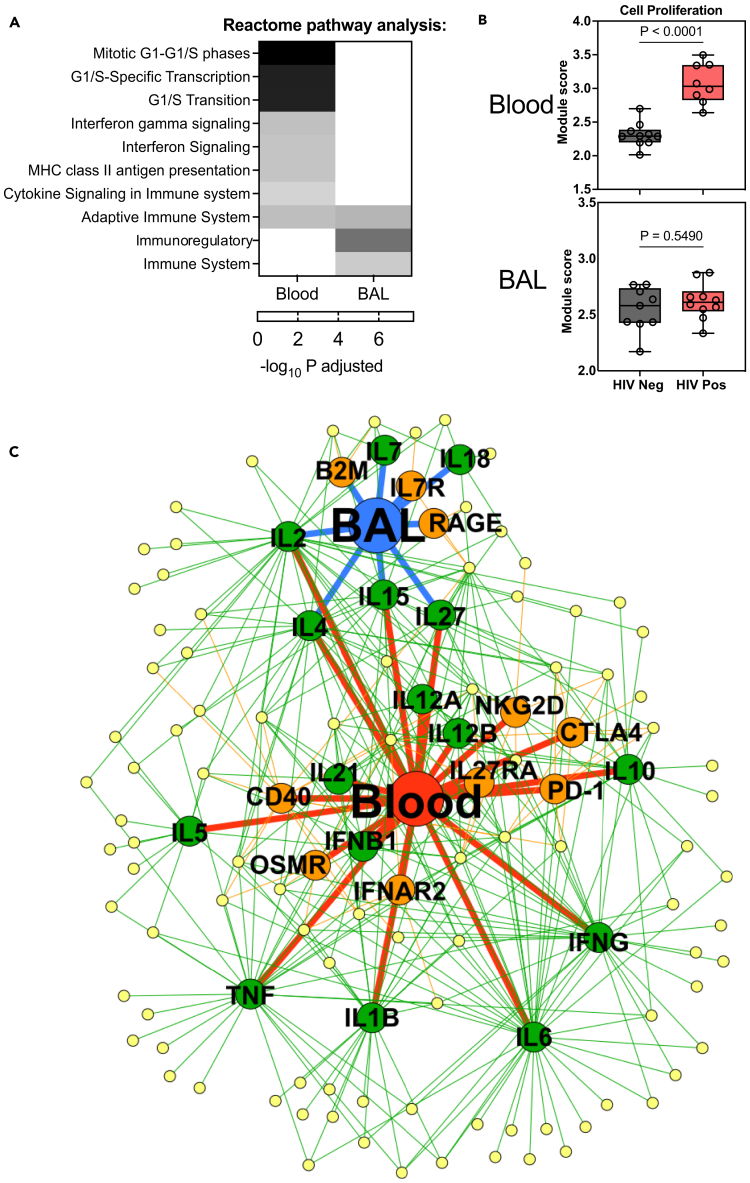
Systems-level dysregulation of immune responses associated with HIV infection in blood and the bronchoalveolar compartment (A) Heatmap showing REACTOME pathway enrichment (white: lowest to black: highest) in the transcriptome in blood (left column) and BAL (right column) samples in PLWH compared to people without HIV. (B) Box (interquartile range) and Whisker (min and max) plot showing expression of T cell proliferation transcriptional response module in blood and BAL compartments in people with (red) and without (gray) HIV with the boxplots showing. (C) Network diagram depicting predicted upstream regulators of genes with increased expression in BAL or blood of PLWH. Yellow nodes represent genes with significantly greater expression in HIV+ compared to HIV− individuals in either BAL (blue node) or blood (red node). Green and orange nodes represent cytokines and transmembrane receptors, respectively, predicted to regulate the expression of genes in the yellow nodes. Edges depict relationship between upstream regulators and the compartments and the differentially expressed genes. IL, interleukin; IFN, interferon; TNF, tumor necrosis factor; OSM, oncostatin M; PD-1, programmed cell death 1; RAGE, receptor for advanced glycation end products; B2M, beta-2 microglobulin.

Systems level analyses of differentially expressed transcripts revealed blood, but not BAL, in HIV was characterized by enrichment of cell proliferation pathways (Figure 2A), elevated expression of *Mki67* gene (Figure 1C), and of an independently derived transcriptional module^12^ corresponding to T cell proliferation (Table S4; Figure 2B). We explored further the immune pathways dysregulated in HIV by predicting the signaling activity of cytokines and transmembrane receptors associated with genes overexpressed in either compartment in the context of HIV (Figure 2C). These data confirmed that untreated HIV infection was associated with widespread immune overactivity, particularly in blood. Predicted immune dysregulation included greater activity of type I and II interferons (IFNs) in blood and of cytokines associated with T cell biology (e.g., interleukin-2 [IL-2], IL-15, and IL-27) in both compartments.

**Figure 3:**
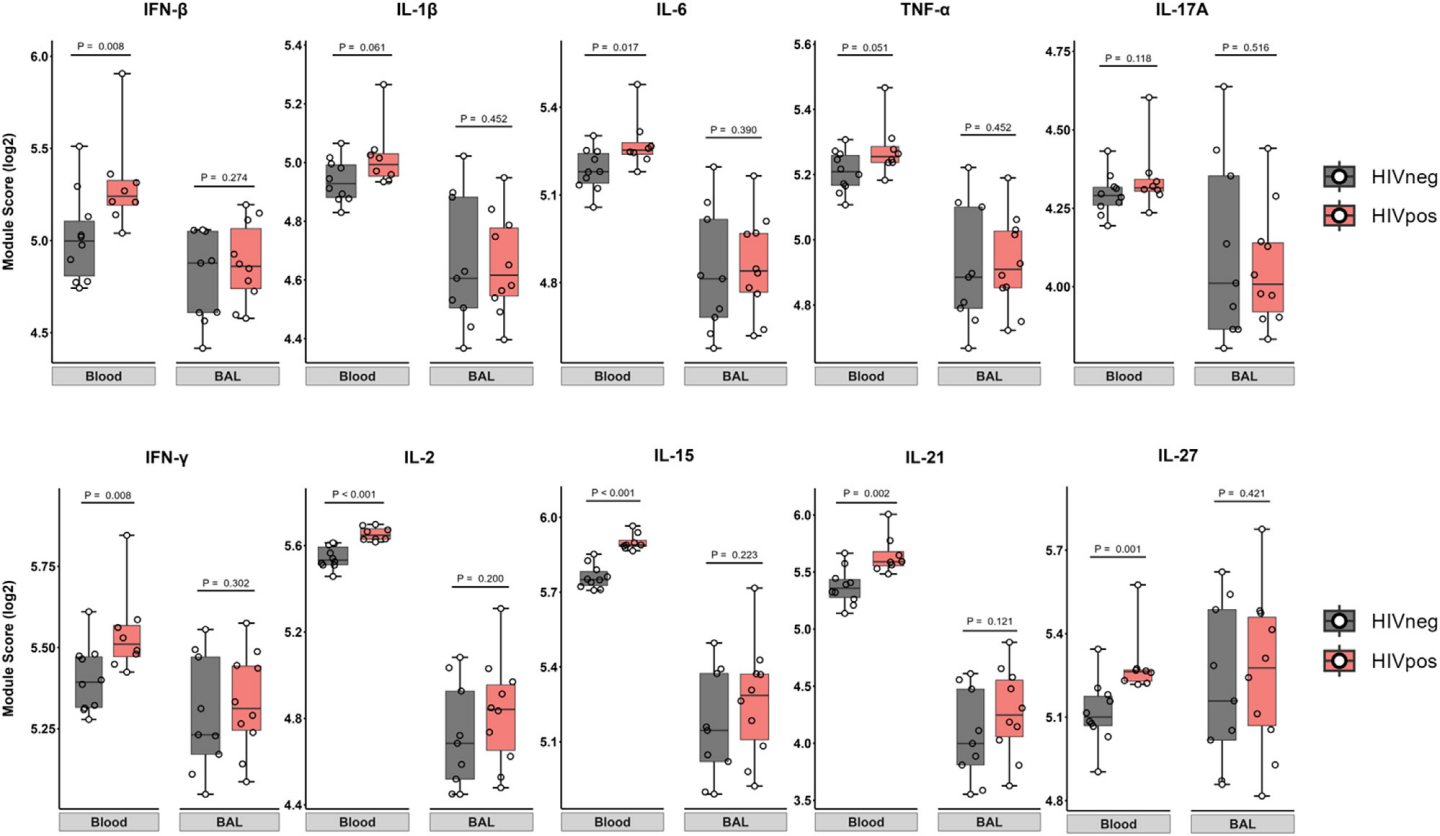
Differential cytokine activity in peripheral blood and BAL compartments associated with HIV infection Box (interquartile range) and whisker (min and max) plots showing expression in the bulk transcriptome of modules reflective of cytokine activity in blood and BAL, stratified by the presence (red) or absence (gray) of HIV infection. Statistical assessments were performed by Mann-Whitney U tests.

To verify this predicted cytokine activity in PLWH, we quantified the expression of independently derived cytokine-inducible transcriptional modules of cell-mediated immunity^13^ in the transcriptional profiles from blood and BAL samples (Figure 3; Table S5). These data confirmed elevated functional activity in blood of both type I and II IFN responses and of cytokines that drive T cell proliferation and activation (IL-2, IL-15, IL-21, and IL-27), as well as more modest increased activity of pleiotropic pro-inflammatory cytokines, such as IL-1β, IL-6, and tumor necrosis factor alpha (TNFα) (Figure 3). Notably, there was no clear enrichment for cytokine activity in BAL samples from PLWH, indicating the differential gene expression observed in this compartment may relate more to the relative enrichment of immune cell subsets than the action of cytokines. Indeed, despite the BAL cellular composition being dominated by alveolar macrophages (Figure 1B), several of the 13 genes overexpressed in PLWH were lymphocyte-associated (Figure 1C, right), and many of these genes showed predictive signaling through Beta-2 microglobulin component of the MHC class I (Figure 2C), suggesting an enrichment of CD8 T cells in BAL in the context of HIV.

### Dysfunctional effector memory CD8 T cells in HIV infection

**Figure 4:**
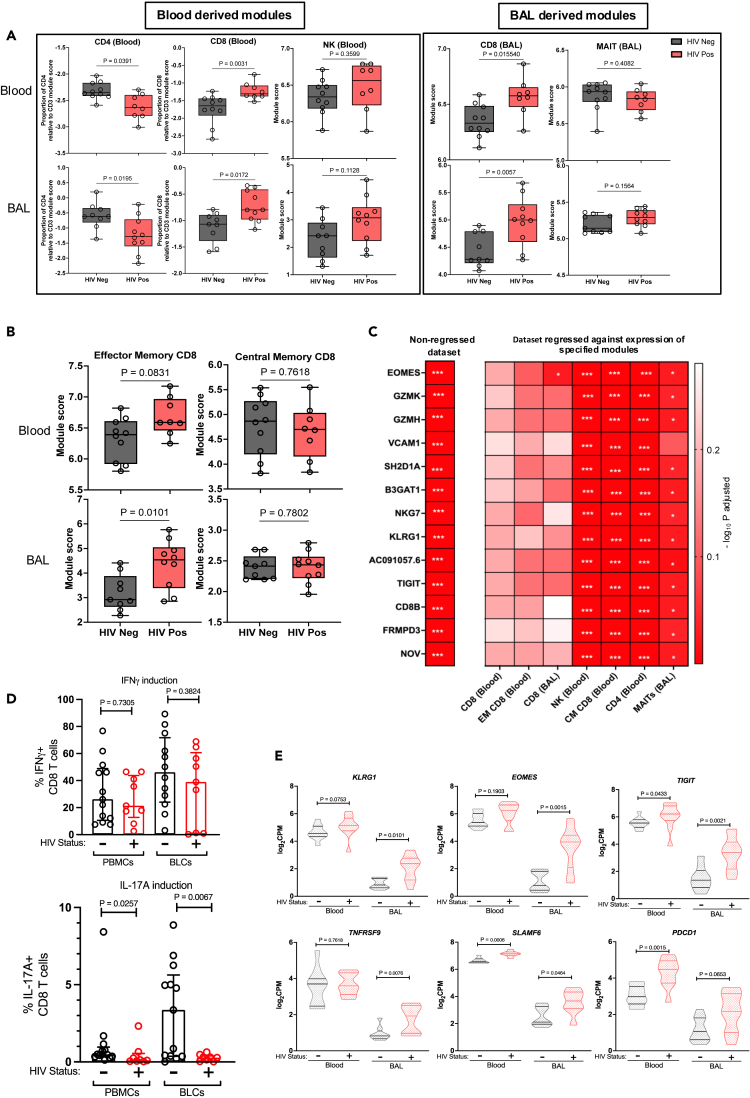
Bronchoalveolar compartment in HIV infection characterized by enrichment of effector memory CD8 T cells deficient in IL-17A production Box (interquartile range) and whisker (min and max) plot illustrating module expression in whole transcriptomics data from (A). Blood-derived (left) and BAL-derived (right) modules and (B) effector and central memory CD8 T cell modules. Statistical assessments were performed by Mann-Whitney U tests. (C) Heatmap representing differential expression of specified genes between PLWH and those without HIV in BAL before (left column) or after linear regression for the expression of specified immune cell transcriptional modules (right columns). *p* values quantified by Mann-Whitney U tests (red = most significant). ∗ <0.05, ∗∗ <0.005, and ∗∗∗ <0.0001. (D) Scatterplot with bars (median) and whiskers (interquartile range) displaying expression of intracellular IFN-γ (top row) and IL-17A (bottom row) by CD8 T cells following PMA-ionomycin stimulation of mononuclear cells from both blood and BAL (*n* = 30, 17 HIV-negative and 13-HIV positive). (E) Violin plots (lines = interquartile range and shape = density) showing multiple immune checkpoint markers transcriptional expression in blood and BAL (left and right, respectively) (*n* = 20, 10 HIV-negative and 10 HIV-positive). Statistical assessments were performed by Mann-Whitney U tests.

Validated transcriptional modules that quantify the relative abundance of immune cells in bulk transcriptomic data (Table S4) demonstrated relative depletion of CD4 T cell transcripts in both blood and BAL in PLWH,^9^ an expansion of CD8 T cells in both compartments, and no differences in natural killer (NK) cell frequency in either compartment (Figure 4A, left). As these validated modules were derived from peripheral blood cells, and might not accurately reflect the transcriptional state of BAL lymphocytes,^9^ we generated an independent set of modules derived from genes differentially expressed in sorted BAL cells (gating strategy in Figure S2). The BAL-derived module specificity was comparable to those from blood, with better discrimination of CD8 T cells from MAIT cells (Figure S3).^14^ These BAL-derived modules (Table S4) confirmed enrichment of CD8 T cells, but not MAIT cells, in both anatomical compartments of PLWH (Figure 4A, right). Flow cytometry confirmed elevated CD8 T cell frequency in blood and BAL of PLWH (Figure S4), providing confirmation that the use of transcriptional modules could quantify cell enrichment from bulk BAL and blood transcriptomes.

Several of the 13 genes with higher expression in BAL of PLWH were associated with CD8 T cell effector functions (*GZMK*, *GZMH*, *B3GAT1*, *NKG7*), and we sought to test the hypothesis that this increased expression reflected an increased frequency of effector memory (EM) CD8 T cells. We generated and validated new transcriptional modules from different CD8 T cell functional states (Figure S5), and application of these to the study samples showed enrichment of CD8 T cells with an EM phenotype in BAL of PLWH, with no differences in central memory (CM) CD8 T cells (Figure 4B). To determine whether EM CD8 T cells were the primary source of significantly upregulated genes in BAL of PLWH, we sought to adjust for differences in cell frequency by performing a linear regression at the whole transcriptome level, controlling for cellular module expression in each sample. This revealed that differential expression of the 13 genes elevated in BAL of PLWH was abrogated by regression against either blood- or BAL-derived CD8 T cell modules or the CD8 EM module, but differential expression was not abrogated by regression against the CD8 CM, CD4, NK, or MAIT cell modules (Figure 4C). We interpreted these data to suggest transcriptomic perturbations in the BAL compartment of people with untreated HIV were attributable to increased numbers of EM CD8 T cells.

Finally, we sought to determine whether the differential frequency of CD8 T cells we had observed in the BAL compartment in HIV was also associated with different functional activity of these cells in HIV. We quantified the production of IFN-γ and IL-17A by both blood and BAL CD8 T cells before and after stimulation with phorbol mysristate acetate (PMA)/ionomycin (Figure S6A; flow cytometry gating strategy). Baseline cytokine expression was low and did not differ by HIV status (Figure S6B), but following stimulation, we observed a lower frequency of CD8 T cells expressing IL-17A in both blood and BAL compartments of PLWH, whereas no such difference was observed for IFN-γ (Figure 4D). Quantification of *KLRG1*, *EOMES*, and *TIGIT* and other genes known to be upregulated in exhausted and functionally inhibited CD8 T cells (*TNFRSF9*, *SLAMF6*, and *PDCD1)*^15^ demonstrated higher expression of these transcripts in either or both compartments of PLWH (Figure 4E). Flow cytometry confirmed higher expression of PD-1 (encoded by the gene *PDCD1*) on blood CD8 T cells in PLWH (Figures S6C and S6D). Overall, these results suggest increased immunoregulatory activity in uncontrolled HIV infection may attenuate IL-17A responses of CD8 T cells in both blood and BAL.

## Discussion

HIV infection increases the risk of respiratory infections including TB.^1^ Using systems-level analysis of the immunobiology of samples from respiratory mucosa in a TB-endemic Southern African context, we demonstrate that uncontrolled HIV infection results in transcriptional changes in the lung’s mucosal compartments that differ from those observed in the blood. The BAL of PLWH is enriched for CD8 T cells transcriptionally consistent with an effector memory phenotype but with elevated expression of immunoregulatory molecules and deficient IL-17A responses upon stimulation. Our data support a model whereby impaired CD8 T cell production of IL-17A may contribute to the susceptibility to secondary bacterial infections in HIV infection.^16^

We extend our earlier report^11^ derived from bulk transcriptomic assessments of BAL cells from a small number of clinical samples, by including a larger number of participants in whom active lung disease was rigorously ruled out and all of whom had evidence of past exposure to *Mtb* but no history of TB disease. Unbiased transcriptional biological assessments revealed in the peripheral blood of PLWH evidence for elevated activity for both type 1 and II IFN pathways, greater T cell proliferation, possibly reflecting a homeostatic response to the depletion of CD4 T cells in HIV infection,^17^ and elevated signaling by IL-2, a key T cell growth factor, IL-15, and IL-27 that can promote the survival and expansion of CD8 T cells.^18^^,^^19^ Although we observed perturbed T cell responses in BAL in PLWH, there was little overlap in differential gene expression associated with HIV infection between blood and BAL. This may reflect discordant immune perturbation in each compartment but also could be driven by differences in the proportions of cells present in these locations.

The relative enrichment for CD8 T cells in BAL of PLWH was evident despite macrophages being the predominant cell type in BAL. The effect of HIV on alveolar macrophage function has been described at the protein^20^ and at transcriptional and epigenetic levels,^21^ but sensitivity to detect these in our dataset may have been precluded by the multicellular, bulk transcriptomic approach taken, as well as the assessment of baseline BAL cells prior to *in vitro* stimulation. Although we were still able to detect HIV-associated depletion of CD4 T cells, confirmed bioinformatically and by flow cytometry, cells that are known to be functionally impaired by HIV,^2^^,^^17^^,^^22^ future work exploring compartment- and immune-cell-specific HIV-induced biological perturbation will require single-cell transcriptomic approaches that were not available in our study. Nevertheless, these technical limitations underline further the significance of HIV-associated changes in CD8 T cells that were detected, making up almost all of the few genes with elevated expression in PLWH. This surprisingly parsimonious number of differentially expressed genes may have been affected by the bioinformatic approaches used but is most likely due to the greater clinical homogeneity of the research bronchoscopy cohort in whom all participants had a positive QuantiFERON (QTF) test and were in a state of systemic and respiratory health, even those with recently diagnosed and yet untreated HIV.

Using modular analyses as an approach to bioinformatically de-convolute whole-genome transcriptomic data, we confirmed the pathway-level analyses and the well-known consequences of HIV infection in depleting CD4 T cells and enriching CD8 T cells in both blood and BAL.^17^ The increased frequency of CD8 T cells in BAL is consistent with reports of lymphatic alveolitis^8^ and was associated with elevated expression of several genes linked to cytotoxicity, such as granzymes H and K, and a transcriptional module reflective of EM CD8 T cells, but not CM CD8 T cells, NK, or MAIT cells. Nevertheless, both blood and BAL CD8 T cells showed diminished IL-17A production after *ex vivo* stimulation, but no impairment in IFN-γ production. This IL-17A defect has been reported in the blood of virally suppressed people with HIV,^23^ but our current study shows that this is also evident in the airways of people with untreated HIV. Taken together, the finding of enriched and functionally altered effector memory CD8 T cells in the BAL of HIV seropositive individuals confirms our previous observations.^11^ This is alongside other reports in the literature^8^^,^^24^ and has also been recently reported in people on long-term antiretroviral therapy (ART),^25^ suggesting this is a reproducible process induced by HIV infection. Moreover, this confirms that the combination of unsupervised and supervised analyses used in our bioinformatic pipeline were sufficiently sensitive to deconvolute compartment-specific transcriptional profiling perturbed by HIV and could also be repurposed for use in large human cohorts in resource-limited setting for assessments of other diseases of the lung mucosa without resorting to more costly single-cell sequencing approaches.

Although IFN-γ is considered an important cytokine in the protection against TB disease,^26^^,^^27^ the frequency and intensity of IFN-γ responses do not correlate with protection from TB disease both following vaccination or after natural TB exposure.^28^^,^^29^^,^^30^^,^^31^ Recent work has indicated that differential IL-17 responses characterize stages of TB infection,^10^ and thus their attenuation by HIV may affect the nature of TB presentation in HIV co-infections. IL-17A is also reported to confer protection against hypervirulent *Mtb* strains such as HN878,^32^ and this may contribute to susceptibility to TB disease in ARV-naïve PLWH.^33^ Active TB is characterized by exaggerated IL-17A responses that may contribute to tissue pathology,^10^ and so our observation of abrogated IL-17A production by CD8 T cells of PLWH may also provide an explanation for why severe chest X-ray abnormalities, such as cavities, are less common in TB patients with advanced HIV.^33^ Notably, we observed the defect in IL-17A production in PLWH to be associated with increased expression of molecules associated with T cell immunomodulation, suggesting an exhausted CD8 T cell phenotype. Indeed, it has been proposed that elevated expression of inhibitory receptors is responsible for diminished IL-17A responses in HIV.^23^ Our findings suggest that modulation of negative regulators of T cell function using targeted biologic agents could be one strategy to restore HIV-induced dysregulated immunity in the lung mucosa.

### Limitations of the study

Our work has several limitations. First, this was a single-center study with a limited sample size that assessed individuals at a single time point. Participants with HIV were all ART-naïve, and we cannot predict if the immune perturbations observed would persist during suppression of HIV replication by ART. We had planned to offer repeat bronchoalveolar lavage to participants after initiation of ART, but this was curtailed by the onset of the COVID-19 pandemic. Our study design purposefully restricted participation to individuals with evidence of latent TB (positive QTF test). This choice was made because in a TB endemic setting such as the one used in our study, a PLWH and QTF-negative population cannot be assumed to be naive to Mtb, especially as our recent data suggest those with negative QTF tests still have anti-*Mtb* antibodies (i.e., reflecting false-negative tests for *Mtb* immune memory).^34^ Nevertheless, this design prevented assessment of the effects of HIV alone in the absence of evidence of latent TB infection. Assessment of unstimulated transcriptomes at bulk cell level precluded assessment of the dynamic nature of responses in response to bacterial stimulation, and *in vitro* stimulations were only performed using non-specific mitogens, meaning that we could not derive the frequency of cytokine-producing *Mtb*-specific CD8 T cells. In addition, bulk transcriptomics prevented definitive conclusions about the cellular origin of differential gene expression observed between PLWH and HIV-negative controls. We were able to attribute cellular origins by regression of gene expression for the relative proportion of different immune cell subsets, but EM and CM CD8 T cell modules could only be derived from blood cell transcriptomes and may have masked specific differences in BAL immune cell biology. We also acknowledge that although we performed analyses from blood and BAL separately, these compartments are unlikely to be fully separate, linked by cell migration and endocrine activity of cytokines, but our study was not able to address this relationship. Additionally, our flow cytometric panel was limited and lacked markers needed to identify tissue-resident cells, especially those within the lung mucosa, and EM and CM CD8 T cells or Gamma-Delta T cells, which are reported major producers of IL-17 in TB infection.^35^ This limited the ability to assess the effects of HIV on these important cells and should be considered for future studies. Finally, we only investigated a limited number of cytokine responses or cytotoxic molecules at the protein level in blood and BAL cells, leaving unanswered whether the observed defects in IL-17A responses are associated with alterations to a larger set of responses in HIV.

### Conclusions

Our study leveraged unique access to both blood and BAL samples of a well-defined cohort of individuals to unmask immune dysregulation associated with uncontrolled HIV infection at the site of pulmonary host-pathogen interactions. We conclude that HIV is associated with divergent transcriptional effects in the blood and bronchoalveolar compartments. Blood was characterized by elevated T cell proliferation and type I/II interferon signaling activity, whereas BAL was enriched for CD8 T cells with an effector memory phenotype but a defect in inducible IL-17A production, associated with increased expression of T cell inhibitory molecules. Future work will need to assess the ability of ART to reverse this immune dysregulation and explore the role of adjunctive immune checkpoint inhibitors to restore mucosal CD8 T cell function and improve immune protection against respiratory infections in HIV.

## Resource availability

### Lead contact

If further information and requests for resources are required, they should be directed to and will be fulfilled by the lead contact, Emily Wong (emily.wong@ahri.org).

### Materials availability

There are no more biological materials to share. The current study sourced transcriptional modules derived from multiple open-access publications. BAL transcriptional modules were generated using transcriptional data generated in this study, and the gene content list for each module is available in Table S4.

### Data and code availability

• The original de-identified human RNA-Seq datasets for whole sample transcriptomics and BAL-sorted immune cell populations (used to generate BAL modules) generated from this study can be found in the Gene Expression Omnibus (GEO) repository as indicated in the key resources table.
• The R code used to calculate the geometric mean expression of modules and perform regression analyses is freely available (https://github.com/innate2adaptive). The gene content of the transcriptional modules generated, derived, and applied to the analyses in this manuscript are available in Table S5.
• Supplementary materials are made available in Supplementary Figures and Supplementary Tables files consisting of data provided by the authors to benefit the reader. The supplementary materials are not copyedited and are the authors' sole responsibility, so questions or comments should be addressed to the corresponding authors. Any additional information required to reanalyze the data reported in this paper is available from the lead contact upon request.

## Acknowledgments

We appreciate the study participants for their participation in the research, all the nurses from Inkosi Albert Luthuli Hospital for their support in acquiring the blood and BAL samples, and the AHRI laboratory biorepository department for handling and distributing the samples. This work was supported by the 10.13039/100000002National Institutes of Health (K08 AI118538) and the Burroughs Wellcome Fund Pathogenesis of Infectious Diseases Award (1022002) and a University College London Global Partnership grant for collaboration between University College London and the Africa Health Research Institute. Additional support was received from the Strategic Health Innovation Partnerships (SHIP) Unit of the South African Medical Research Council with funds received from the South African Department of Science and Innovation as part of a bilateral research collaboration agreement with the Government of India. Supplementary support was received through the Sub-Saharan African Network for TB/HIV Research Excellence (SANTHE), a DELTAS Africa Initiative (grant no. DEL-15-006). The DELTAS Africa Initiative is an independent funding scheme of the African Academy of Sciences Alliance for Accelerating Excellence in Science in Africa (AESA) and supported by the New Partnership for Africa’s Development Planning and Coordinating Agency (NEPAD Agency) with funding from the 10.13039/100010269Wellcome Trust [grant no. 107752/Z/15/Z] and the 10.13039/100013986UK Government. Other support was acquired through the South Africa Research Chairs Initiative, 10.13039/501100022674The Victor Daitz Foundation, a Burroughs-Wellcome Fund/American Society of Tropical Medicine and Hygiene fellowship (EBW). GP’s time was funded by the UCLH NIHR Biomedical Research Centre. Research reported in this publication was also supported by the 10.13039/100000049National Institute on Aging and Infectious of the National Institutes of Health under award number [UM1 AI068618] (E.A.N.). The content is solely the responsibility of the authors and does not necessarily represent the official views of the National Institutes of Health. This research was funded in whole, or in part, by Wellcome Strategic Core award: [227167/A/23/Z]. For the purpose of open access, the author has applied a CC BY public copyright license to any Author Accepted Manuscript version arising from this submission.

## Author contributions

Conceptualization and methodology, M.Mthembu., T.N., G.P., and E.W.; investigation, M.Mthembu., S.K., and A.N.; writing—original draft, M.Mthembu., G.P., and E.W.; writing—review & editing, M.Mthembu., T.N., G.P., and E.W.; formal analysis, M.Mthembu., H.C., V.V., J.S., T.N., G.P., and E.W.; funding acquisition, M.Mthembu., T.N., G.P., and E.W.; resources, K.N., D.F.K., P.M., M.Mitha., Z.M., and F.K.; supervision, T.N., G.P., and E.W. All authors viewed and approved the final manuscript version.

## Declaration of interests

No conflicts of interest were reported by any of the co-authors.

## STAR★Methods

### Key resources table

**Table:** 

REAGENT or RESOURCE	SOURCE	IDENTIFIER
**Antibodies**		

Brilliant Violet 650™ anti-human CD3, clone: OKT3	BioLegend	Cat# 317324; RRID: AB_2563352
Brilliant Violet 711™ anti-human CD4, clone: OKT4	BioLegend	Cat# 317440; RRID: AB_2562912
APC-Cyanine7™ anti-human CD14, clone: HCD14	BioLegend	Cat# 325620; RRID: AB_830693
Brilliant Violet 421™ anti-human PD-1, clone: EH12.1	BD Biosciences	Cat# 562516; RRID: AB_11153482
Brilliant Violet 785™ anti-human TIM-3, clone: F38-2E2	BioLegend	Cat# 345031; RRID: AB_2565833
C8 Monoclonal antibody (3B5) PE Texas Red, clone: 3B5	Invitrogen	Cat# MHCD0817; RRID: AB_10372359
Alexa Flour 700 anti-human CD3, clone: UCHT1	BioLegend	Cat# 300424; RRID: AB_493741
APC/Cyanine7 anti-human CD8, clone: SK1	BioLegend	Cat# 344714; RRID: AB_2044006
Alexa Flour 647 anti-human granzyme B, clone: GB11	BioLegend	Cat# 515406; RRID: AB_2566333
Brilliant Violet 421™ anti-human IL-17A, clone: BL168	BioLegend	Cat# 512322; RRID: AB_11218604
PE/Dazzle 549 anti-human IFN-γ, clone: 4S.B3	BioLegend	Cat# 502546; RRID: AB_2563627
MR1-6-FP (negative control)	NIH Tetramer Core Facility	–
MR1-5-OP-RU (mait-specific tetramer)	NIH Tetramer Core Facility	–

**Biological samples**		

Whole Blood Samples	This Study	N/A
Whole BAL samples	This Study	N/A
BAL mononuclear cells (BLCs)	This Study	N/A
Peripheral blood mononuclear cells (PBMCs)	This Study	N/A

**Chemicals, peptides, and recombinant proteins**		

Phorbol 12-myristate 13-acetate (PMA)	Sigma Aldrich	Cat no: 16561-29-8
Ionomycin	Sigma Aldrich	Cat no: 56092-82-1

**Critical commercial assays**		

QuantiFERON-TB Gold Plus 2 Plates ELISA Kit	QIAGEN	Cat no: 622130
QuantiFERON-TB Gold Plus Blood Collection Tubes	QIAGEN	Cat no: 622536
Live/Dead Fixable Aqua Dead Cell Stain Kit	ThermoFisher Scientific	Cat no: L34957
Nextera XT DNA Sample Preparation Kit, 96 samples	Illumina	Cat no: FC-131-1096
Nextera XT Index Kit, 96 indices, 384 samples	Illumina	Cat no: FC-131-1002
PAXgene Blood RNA kit	QIAGEN	Cat no: 762174
PAXgene Blood RNA Tubes	BD Biosciences	Cat no: 762165
RNeasy Micro kit	QIAGEN	Cat no: E7490
NEBNext Poly(A) Mrna Magnetic Isolation Module	New England BioLabs	Cat no: E76005
NEBNext Multiplex Oligos for illumina	New England BioLabs	Cat no: E7490
NEBNext Ultra RNA Library Prep kit	New England BioLabs	Cat no: E7530

**Deposited data**		

Dysfunctional effector memory CD8 T cells in the bronchoalveolar compartment of people living with HIV	GEO (Whole transcriptomics)	GSE230738
Dysfunctional effector memory CD8 T cells in the bronchoalveolar compartment of people living with HIV	GEO (BAL-mini-population transcriptomics)	GSE231628

**Oligonucleotides**		

Template switching oligonucleotides	IDT DNA	Customized
Reverse transcription DNA oligonucleotides	IDT DNA	Customized

**Software and algorithms**		

Prism 10.2.3	GraphPad	NA
FlowJo 10.9.0	BD Biosciences	NA
Ingenuity Pathway Analysis version: 111725566	QIAGEN	https://digitalinsights.qiagen.com/products-overview/discovery-insights-portfolio/analysis-and-visualization/qiagen-ipa/
Transcriptional module R scripts	GitHub Biorepository	https://github.com/MJMurray1/MDIScoring)
RNASeqPipelineR (alignment, quantification and annotation of the RNA sequencing data)	GitHub Biorepository	https://github.com/RGLab/RNASeqPipelineR
STAR (v2.4.2a)	GitHub Biorepository	https://github.com/alexdobin/STAR
SARTools	GitHub Biorepository	https://github.com/PF2-pasteur-fr/SARTools
InnateDB (Gene Ontology Analysis)	InnateBD	https://www.innatedb.com/redirect.do?go=batchGo

**Others**		

A HaemAtlas: characterizing gene expression in differentiated human blood cells	ArrayExpress	E-TABM-633, https://doi.org/10.1182/blood-2008-06-162958
Landscape of stimulation-responsive chromatin across diverse human immune cells	NCBI GEO	GSE117164, https://doi.org/10.1038/s41588-019-0505-9
IL-12 selectively programs effector pathways that are stably expressed in human CD8^+^ effector memory T cells *in vivo*	NCBI GEO	GSE27337, https://doi.org/10.1182/blood-2011-05-357111
A common transcriptomic program acquired in the thymus defines tissue residency of MAIT and NKT subsets	ArrayExpress	E-MTAB-7143, https://doi.org/10.1084/jem.20181483
Rapid synchronous type 1 IFN and virus-specific T cell responses characterize first wave non-severe SARS-CoV-2 infections.	ArrayExpress	E-MTAB-10022, https://doi.org/10.1016/j.xcrm.2022.100557

### Experimental model and study participant details

#### Study subjects

We recruited otherwise healthy adults (ages 18–60 years) grouped by HIV serostatus, from a research bronchoscopy cohort previously described by Muema et al., 2020^11^ and Khuzwayo et al., 2021^36^ in Durban, South Africa. Inclusion criteria included a positive QuantiFERON-TB Gold Plus for all participants and, for HIV seropositive individuals, no previous exposure to ART. People with newly diagnosed HIV were referred for immediate antiretroviral therapy according to South African Department of Health guidelines. Exclusion criteria included a prior diagnosis of TB disease, abnormal chest X-ray, any other co-morbid disease, any current or prior tobacco use, and current pregnancy.

#### Ethical approval

Bronchoalveolar lavage (BAL) fluid and paired peripheral blood samples were sourced from a research bronchoscopy study called the Phefumula study, based at Africa Health Research Institute (AHRI) in Durban, South Africa. This study was approved by the University of KwaZulu-Natal Biomedical Research Ethics Committee (BREC; reference numbers BF503/15 and BE037/12) and the Partners Institutional Review Board.

### Method details

#### Sample acquisition

Paired blood and BAL samples were obtained from all study participants. Evaluation for latent TB status was performed by QuantiFERON-TB Gold Plus (QTF-Plus, Qiagen), HIV status was determined using 4^th^ generation HIV antibody/antigen Enzyme Linked-Immunosorbent Assay (ELISA) testing, HIV RNA quantitative viral load and CD4 T cell count were also quantified. A chest X-ray and sputum GeneXpert were also performed to exclude active TB cases. People who were either HIV-negative or HIV-positive and ART-naïve, had a positive QTF result, and whose blood test met safety criteria for research bronchoscopy (hemoglobin >10 g/dL, platelet count >150 and international normalized ratio (INR) > 1) were consented to participate in this research bronchoscopy study. On the day of the bronchoscopy, a paired peripheral blood draw was collected. The research bronchoscopy procedure has been described previously^37^; briefly after administration of topical lidocaine to the vocal cords and midazolam, the pulmonologist wedged the bronchoscope in the right middle lobe, instilled 200 mL of normal saline and collected lavage fluid for processing.

#### Sample processing

A portion of whole blood and BAL samples were assessed for differential cell count using histochemistry and compound microscopy to count for alveolar macrophages/monocytes, eosinophils, lymphocytes and neutrophils per sample type (Figure S1). The collected paired blood and BAL samples were then transported to the research laboratory in less than 2 h for processing. PAXGene blood collection tubes were immediately frozen. Peripheral blood mononuclear cells (PBMCs) were isolated from the bloods using the standard Histopaque (Sigma-Aldrich) gradient centrifugation method. A 1.5 mL portion of the whole BAL samples was centrifuged (1500 g × 10 min), and the pellet was resuspended in 100 μL RNALater stabilizing reagent (Sigma-Adrich) and frozen at −80°C. The remaining BAL sample was then filtered through a 40 μm filter, centrifuged (1500 g × 10 min) and the cell pellet resuspended in 10% of the sample in saline buffer volume with completed RPMI media (RPMI media supplemented with 5% fetal bovine serum, 1% penicillin/streptomycin, 1% HEPES buffer and 1% amphotericin). When sufficient live mononuclear cells were available from blood and BAL samples, they were subjected to (Phorbol 12-myristate 13-acetate (PMA) (25 ng/mL)/Ionomycin (500 ng/mL)) stimulation for 6 h at 37°C in 96-well microplates and monoclonal antibody staining.

#### Preparation of RNA-seq libraries and sequencing

##### Whole compartment

Ten pairs of blood and BAL samples from HIV-negative people and 10 pairs of blood and BAL samples from HIV-positive people (Table 1) were randomly selected from the enrolled participants with >90% cell viability from flow cytometry data and used to prepare RNA-Seq libraries. Preserved blood and BAL samples were thawed from −80°C storage to room temperature (RT). RNA was extracted from the Blood PAXgene tubes using PaXgene Blood RNA kit (Qiagen) according to the manufacturer’s recommendations. Briefly, PAXgene blood samples rested at RT for 2 h to facilitate cell lysis and then were centrifuged and the pellet was washed. Proteinase K was used to remove proteins from the samples and the sample passed through a Shredder spin column and DNAse I treated to homogenize and remove any genomic DNA. Filtrate was subsequently transferred into the RNA Spin column to isolate the total RNA in each sample. RNA extraction from the BAL samples was done using RNAEasy Micro Kit (Qiagen) following the manufacturers protocol. Briefly, the BAL cells in RNALater reagent were pelleted and resuspended in 1% β-mercaptoethanol RLT lysis buffer (Qiagen) and RNA extraction and continued with extraction as above. The concentration and integrity of the extracted RNA were assessed using NanoDrop Lite Spectrophotometer (Fisher Scientific) and 4200 TapeStation System (Agilent Technologies), respectively. We excluded samples with RNA Integrity (RIN) values < 7. RNA-Seq libraries were prepared as described by Muema et al., 2020.^11^ Briefly, RNA from both blood and BAL samples was enriched for messenger RNA using the NEBNext Poly(A) mRNA Magnetic Isolation Module (New England Biolabs). Libraries were prepared using NEBNext Ultra RNA Library Prep Kit for Illumina (New England Biolabs) and barcoded using NEBNext Multiplex Oligos for Illumina kit (New England Biolabs). Library quality was assessed using the DNA TapeStation (Agilent). RNA library concentrations were determined using a Qubit dsDNA assay kit (Thermo Fisher). A pool of barcoded libraries with equal molarities was then sequenced on an Illumina NextSeq 500 platform targeting 10 million reads per sample. After an initial sequencing run that included all samples, selected samples were re-sequenced to achieve goal sequencing depth.

##### Sorted cell populations

In participants with sufficient live cells from BAL compartments, adherent and non-adherent cells were separated by plastic adherence on 6 well plates (CLS3335) at 37°C for 1h. Non-adherent cells were stained using Live/Dead Fixable Aqua (Life Technologies, L34957), CD3-BV650 (BioLegend, 317324), CD4-BV711 (BioLegend, 317440), CD14-APC-Cy7 (BioLegend, 325620), PD-1-BV421 (BD Biosciences, 562516), TIM-3-BV785 (BioLegend, 345031) and CD8-PE Texas Red (Invitrogen, MHCD0817). Adherent cells were scrapped from the plastic. Both samples were acquired on a BD FACSAria III (BD Biosciences) cell sorter to isolate CD4 T cells (defined as single, live, lymphocytes, CD3^+^, CD4^+^), CD8 T cells (defined as single, live, lymphocytes, CD3^+^, CD8^+^), MAIT cells (defined as single, live, lymphocytes, CD3^+^, CD4^−^, MR1-5OP-RU tetramer+), monocytes (defined as single, live, lymphocytes, CD3^−^, CD14^+^) and B-cells (defined as single, live, lymphocytes, CD3^−^, CD14^−^, CD19^+^) and alveolar macrophages (sourced from unstained adherent cells, defined by size and complexity using flow cytometry). The gating and sorting (done for some BAL samples from PLWH) strategy is shown in Figure S2. The purity of sorted cells was confirmed to be >95% for a test sample. Using single-cell sorting mode, ‘mini-populations’ of 100 cells were collected into 50 μL of RLT buffer (Qiagen) with 1% β-mercaptoethanol and subsequently stored at −80°C. Subsequent RNA isolation and library preparation from these samples was conducted using Smart-Seq and Smart-SeqII approaches as described in Trombetta et al., 2014.^38^ Briefly, RNA from lysed mini-populations were isolated using SPRI paramagnetic bead technology (RNAClean XP, Beckman Coulter), and cDNA was prepared by targeting RNA with poly-A tail ensuring reverse transcription of mRNA using Maxima First Strand cDNA Synthesis Kit (Thermo Fisher Scientific, K1641). Whole transcriptome amplification was performed and cleaned using AMPure XP beads (Beckman Coulter). RNA libraries were then prepared using Nextera XT library preparation kit (Illumina).

Library quality was assessed using the DNA TapeStation (Agilent). RNA library concentrations were determined using a Qubit dsDNA assay kit (Thermo Fisher). A pool of barcoded libraries with equal molarities was sequenced on an Illumina NextSeq 500 platform. After an initial sequencing run that included all samples, selected samples were re-sequenced to achieve goal sequencing depth.

#### Transcriptomic data extraction and analyses

The RNASeqPipelineR package (https://github.com/RGLab/RNASeqPipelineR) was used to perform alignment, quantification and annotation of the RNA sequencing data (RNA-seq) from the whole samples and sorted immune cells from BAL. The RNA-seq data were aligned against the human genome (hg38) using STAR (v2.4.2a),^39^ and gene expression quantification was performed using RSEM (v1.2.22).^40^ Genes with less than 15 nonzero read counts were discarded, leaving 19,720 expressed genes for the analysis. Libraries (samples) with less than 500,000 reads; 10,000 detected genes; an alignment rate <75% and an exon rate <50%; and clear sample annotation were confirmed using NGSCheckMate.

For whole compartment samples from blood and BAL, we performed principal component analyses^10^ and whole compartment gene expression profiles were deconvoluted using CIBERSORT to provide an estimation of immune cell abundance.^41^ Differential gene expression of RNA-Seq data using the package SARTools, a DESeq2 R pipeline, using a false discovery rate (FDR) of 0.05.^42^ Pathway analysis of the differentially upregulated genes from the whole compartment samples was performed using the Reactome database via InnateDB^43^ (significantly enriched genes were defined by fold change >1 or < −1 and FDR q value <0.05 or pathways by *p* values adjusted using Benjamini-Hochberg correction), and visualized as network diagrams in Gephi v0.10.1. Ingenuity Pathway Analysis (Qiagen) was used to predict upstream cytokine and transmembrane receptors that regulate the expression of genes, with significant regulators determined by *p* values <0.01.

#### Transcriptional module analyses

We derived the expression of transcriptional modules from the geometric mean expression of all constituent genes within a module, as previously described.^9^

We utilized previously validated transcriptional modules reflective of T cell proliferation and tissue cytokine activity.^12^^,^^13^ For blood CD4 T cells, CD8 T cells, and NK cell modules, we utilized those derived from the bulk transcriptome of purified immune cells^44^ or blood samples, which possess the greatest sensitivity and specificity for cognate purified cell types^9^^,^^29^ and have been validated to reflect relative immune cell frequency *in vivo*^9^^,^^45^ (provided in Tables S4 and S5). Transcriptional modules representing immune cells in BAL were derived from differentially expressed genes between sorted immune cell populations using the DESeq2 R pipeline package, SARTools as above. For each cell type of interest, module constituent genes were derived from genes with significantly elevated expression in the cognate cell relative to specific other cell types using an FDR of 0.05 (Table S4). CD4 T cells were compared to CD8 T cells and MAIT cells, while the CD8 T cells were compared to CD4 T and MAIT cells and the rest of the prepared modules were defined by comparing cognate cell type relative to all others (e.g., CD14^+^ monocytes compared to CD4, CD8 T-cells, MAITs and alveolar macrophages). The sensitivity and specificity of these modules were internally validated from the BAL transcriptome from which they were derived (Figure S3A) and externally validated using an independent dataset of sorted immune cell types (Figure S3B).

Transcriptional modules reflecting the biology of central and effector memory (CM and EM, respectively) CD8 T cells were derived from CD8 T cells purified on the basis of CCR7 and CXCR3 expression, with CCR7^hi^CXCR3^lo^ representing CM and CCR7^lo^CXCR3^hi^ EM CD8 T cells.^46^ We focused on genes that were differentially expressed between CM and EM populations (2-sided t-test, *p* < 0.01 uncorrected for multiple testing) and >10-fold expressed between the 2 groups. Genes that fulfilled these criteria were used to compose CD8 CM and EM transcriptional modules. Their specificity was initially determined by internal validation of expression scores on the dataset of module origin (GSE27337) and then using an independent external dataset (GSE117164) of purified CD8 T cell subsets and NK cells.^47^ These revealed high sensitivity and specificity of the CM and EM transcriptional modules for their cognate cell types (Figure S5).

#### Transcriptional module linear regression

To adjust for the contribution of different immune cell type frequencies on differential gene expression in the BAL transcriptome, a linear regression was performed for each gene’s expression matrix using immune cell’s module expression score to generate a vector of cell counts. The slope and intercept of this vector were used to predict the expression of all genes, and thus calculate residuals for each gene in the matrix for all samples. The expression matrix of residuals generated was then used for differential gene expression and immune module quantification as above.

#### Flow cytometry

Mononuclear cells from both blood and BAL samples were counted and viability was assessed and confirmed to be >90% using trypan blue (Sigma-Aldrich). To assess cell lineage distribution, phenotypes, and functionality of immune cells in blood and BAL compartments with HIV infection we stained mononuclear cells using 2 panels of fluorescently labeled monoclonal antibodies. Panel 1 (phenotypic panel): Same as the sorting panel above. Panel 2 (intracellular cytokine staining panel): Live/dead-amcyan (Life Technologies), CD3-Alexa700 (BioLegend, 300424), CD4-BV711 (BioLegend, 317440), CD8-APC-Cy7 (BioLegend, 344714), granzyme b-Alexa647 (BioLegend, 515406), IFN-γ-PE/Dazzle 594 (BioLegend, 502546), IL-17A-BV421 (BioLegend, 512322). Staining using Panel 2 was done after PMA/Ionomycin (25/500 ng/mL) stimulation. The BDFACSAria III (BD Biosciences) flow cytometer was used to acquire samples. Compensation and rainbow beads were used as standards to minimize day-to-day variability. FlowJo v10.8.1 (FlowJo, LLC) was used for flow cytometry analysis. Comparisons in the flow cytometry data between HIV-negative and HIV-positive people were evaluated using Mann-Whitney U test.

### Quantification and statistical analysis

Statistical analyses were performed using R v4.1.2, Prism v10.3.2 (GraphPad), and FlowJo 10.9.0 using tests described in the figure legends, results, and method detail sections above. Where statistical tests are presented as asterisks, the asterisks are defined in each relevant figure legend, together with the name of the statistical test used.

## Supplemental information

Document S1. Figures S1–S6

Table S1. Demographic and clinical characteristics of the enrolled participants in larger research bronchoscopy cohort

Table S2. Differentially upregulated and downregulated genes in PLWH blood samples

Table S3. Differentially upregulated and downregulated genes in PLWH BAL samples

Table S4. Immune cell subset and proliferation transcriptional modules (bracketed labels indicate compartment source of transcriptional module)

Table S5. Cytokine activity transcriptional modules
