# Supplementary material for: Dysfunctional bronchoalveolar effector memory CD8+ T cells in tuberculosis-exposed people living with antiretroviral-naïve HIV infection
Source: iScience. 2024 Oct 16;27(11):111137. doi: 10.1016/j.isci.2024.111137 (PMC11575450; doi:10.1016/j.isci.2024.111137)
Supplement: Document S1. Figures S1–S6 [file mmc1.pdf]

iScience, Volume *27*

Supplemental information

Dysfunctional bronchoalveolar effector memory $CD8^+$ T cells in tuberculosis-exposed people living with antiretroviral-naïve HIV infection

Maphe Mthembu, Helgard Claassen, Sharon Khuzwayo, Valentin Voillet, Anneta Naidoo, Jule S. Spillner, Kennedy Nyamande, Dilshaad Fakey Khan, Priya Maharaj, Mohammed Mitha, Zoey Mhlane, Farina Karim, Erica Andersen-Nissen, Thumbi Ndung'u, Gabriele Pollara, and Emily B. Wong

Supplementary Figures:

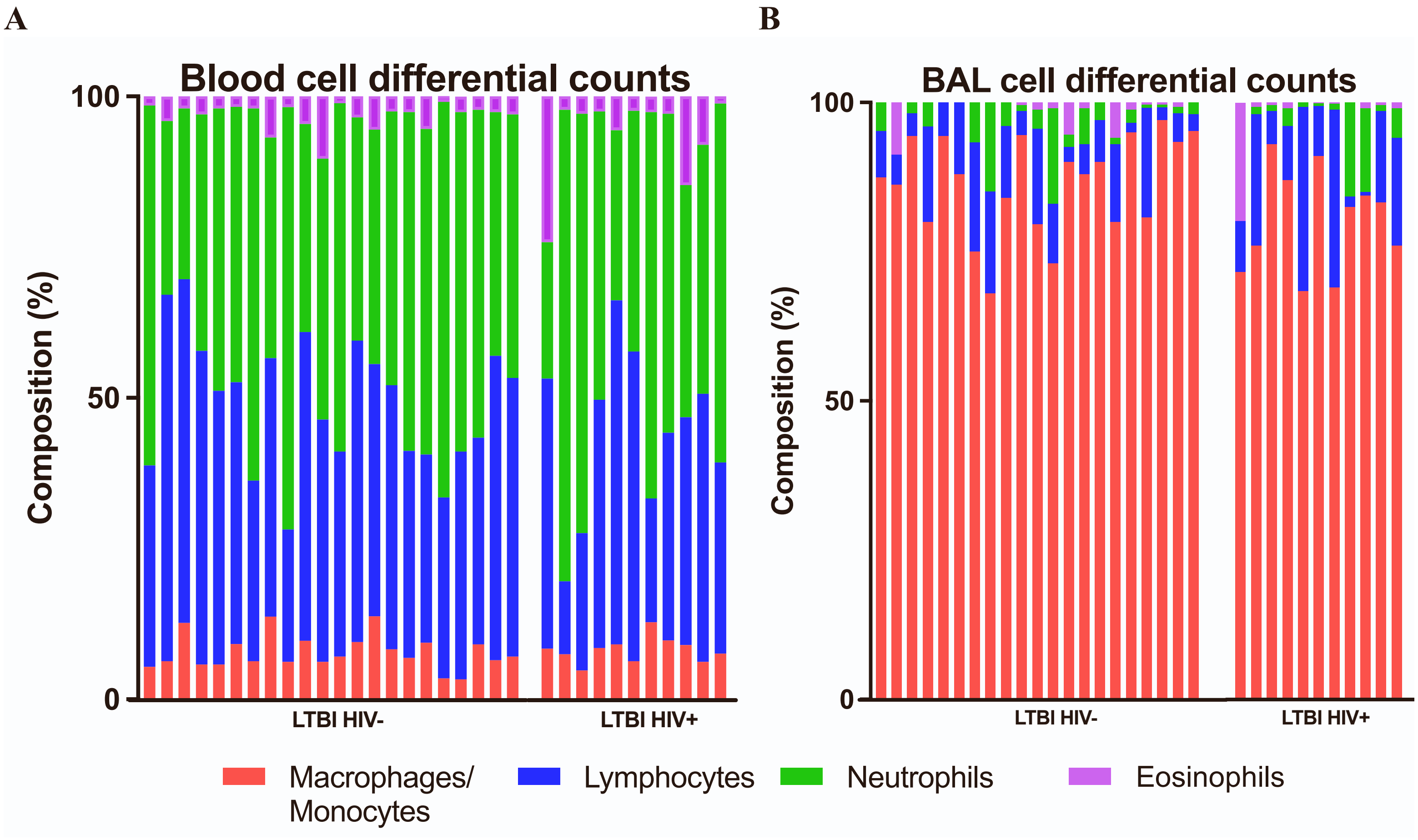

Supplementary Figure 1: Differential cell counts proportions showing the distribution of the major immune cell types in blood and Bronchoalveolar lavage (BAL) compartment. Proportions of different immune cells per participant (column) separated by HIV status on the x-axis in blood (A) and BAL (B) compartments. Of note, 23 of the samples included in this figure were originally included in the preliminary work by Muema et al. 2020. We have included these data here to show consistency in the relative cell proportions, but none of the samples from this previously published work were used in the transcriptional analyses included in this manuscript.

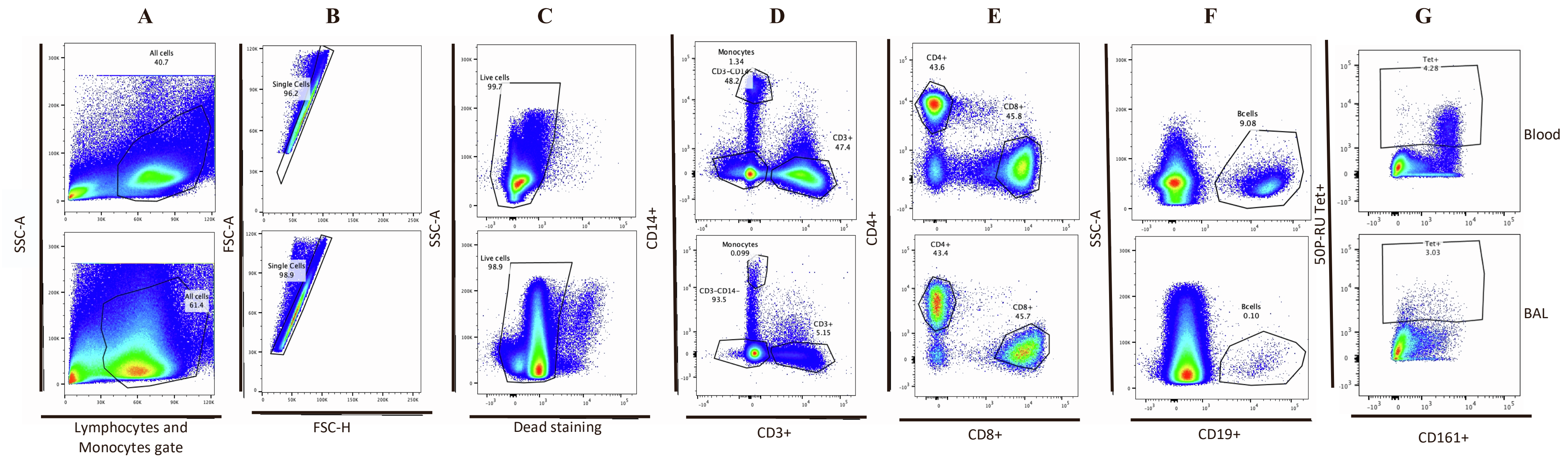

Supplementary Figure 2: Flow cytometry gating strategy for confirmation of immune cell subsets. A. side-scatter-area (SSC-A) vs forward-scatter-Area (FSC-A) are plotted showing the expanded lymphocyte gate which also includes larger-sized monocytes. **B.** FSC-A vs. FSC-Height (-H) are plotted to exclude clustered cells and select for single cells. **C.** dead cell exclusion gate. **D.** CD14+ monocytes, CD3+ T-cells and those that are double negative for CD14 and CD3. **E.** Use cells from the CD3+ T-cells gate to select CD4+ and CD8+ T-cells. **F.** Use double negative cells from the double negative gate and select CD19+ B-cells. **G.** Cells from CD8+ T cells gate are plotted to then gate for 50P-RU Tet+ MAIT cells and simultaneously display nature CD161 expression nature of the gated MAITs. This gating strategy shows representative gates from stained mononuclear cells from both blood: top row and BAL: bottom row.

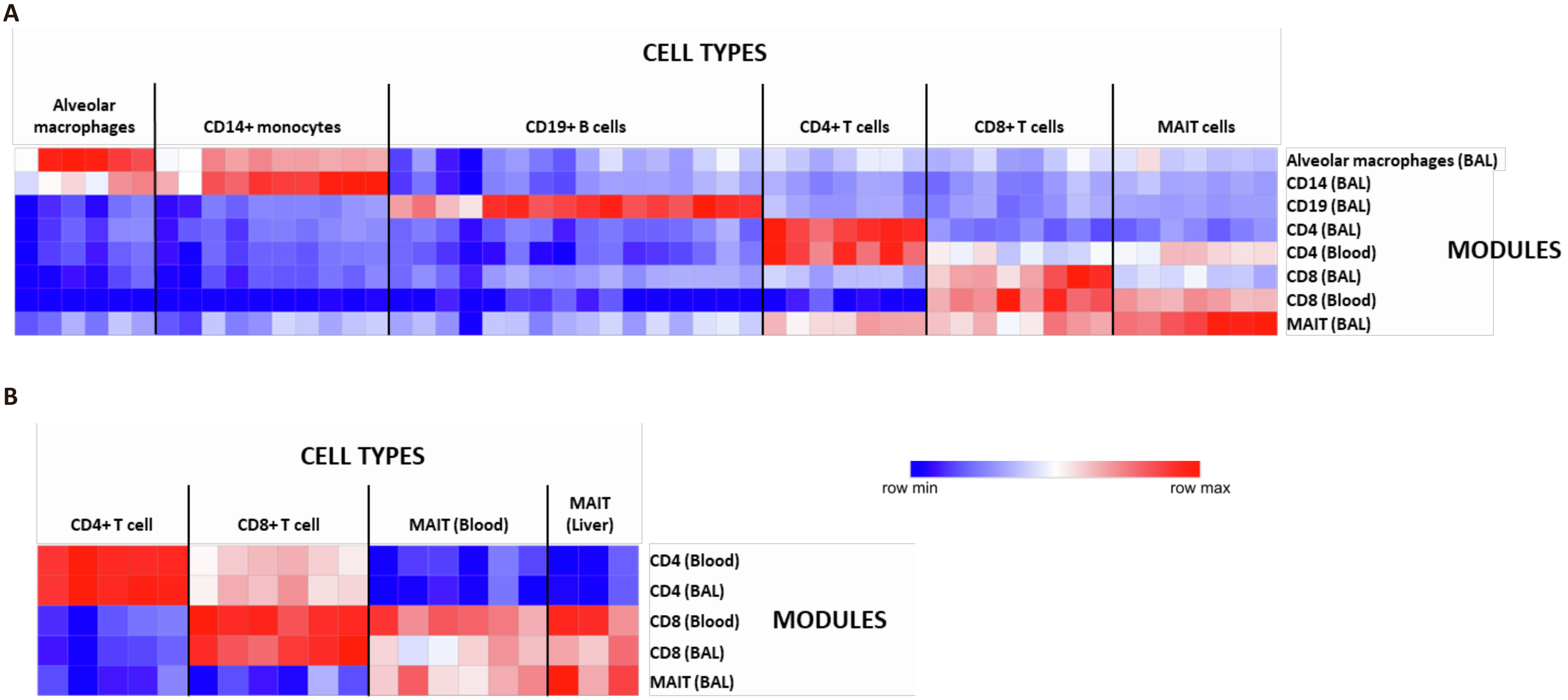

Supplementary Figure 3: Assessment of sensitivity and specificity of Bronchoalveolar Lavage (BAL)-derived immune cell modules. The expression of transcriptional modules derived from flow cytometry sorted immune BAL cells in (A) BAL samples in HIV-positive individuals in the current cohort and (B) sorted CD4, CD8 and MAIT cells from ArrayExpress dataset E-MTAB-7143.

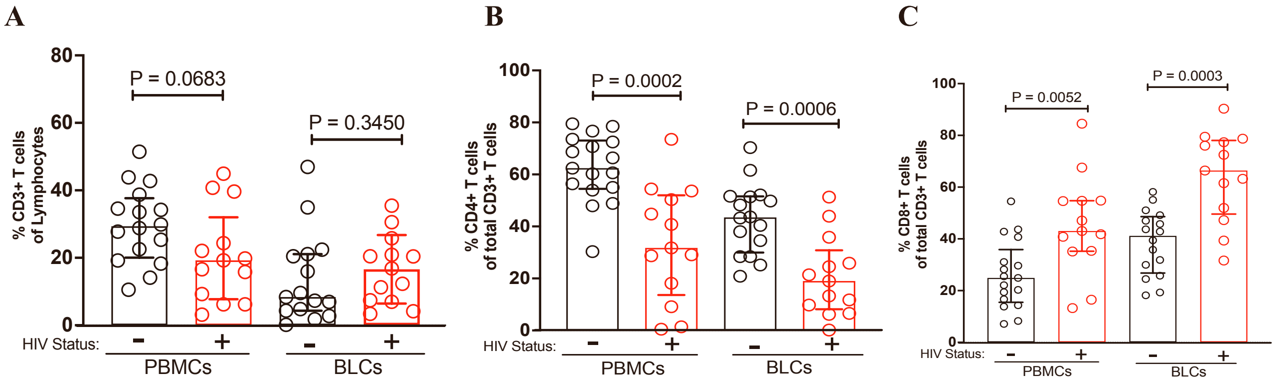

Supplementary Figure 4: Enumeration of immune cells in Peripheral Blood Mononuclear Cells (PBMCs) and Bronchoalveolar Lavage mononuclear Cells (BLCs) in PLWH and those without HIV. Scatter plots with bars (median) and whiskers (IQR) showing quantification of (A) T cells by surface expression of CD3, (B) CD4 T cells by surface expression of CD3 and CD4, and (C) CD8 T cells by expression of CD3 and CD8.

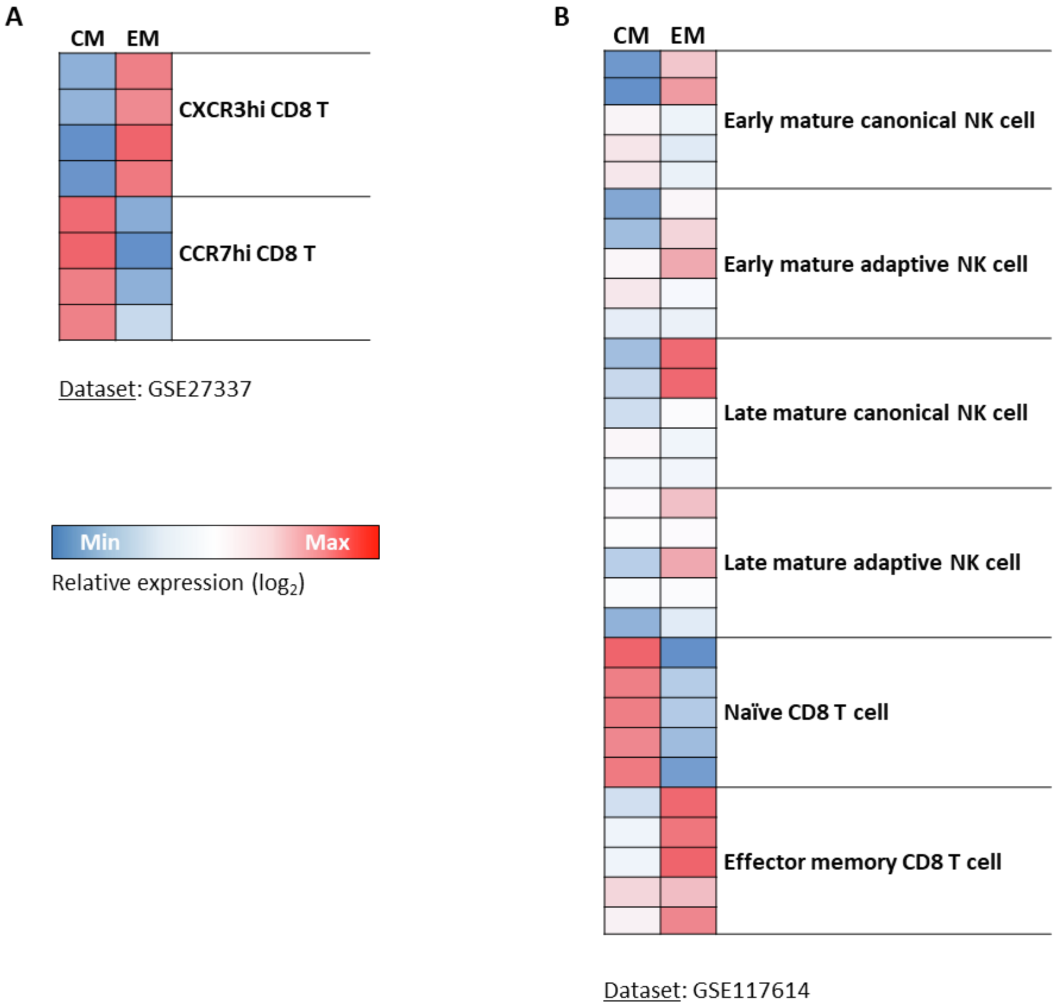

Supplementary Figure 5: Assessment of sensitivity and specificity of central memory (CM) and effector (EM) CD8 T cell modules. The expression of CM and EM CD8 T-cell transcriptional modules in (A) CXRC3hi or CCR7hi CD8 T cells representing EM and CM cells respectively (GEO Omnibus dataset GSE27337) and (B) sorted NK cell and CD8 T cells (GEO Omnibus dataset GSE117614).

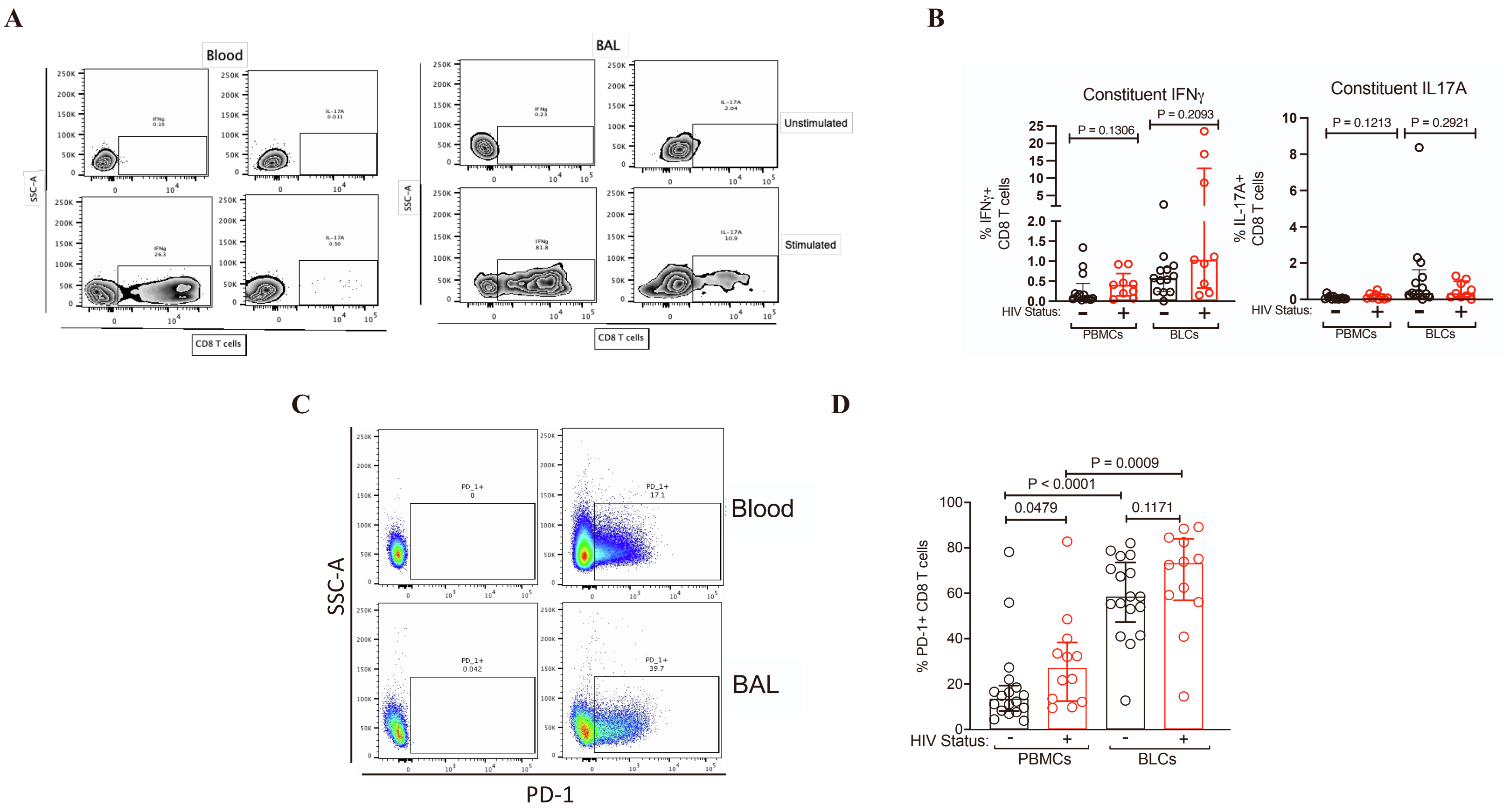

Supplementary Figure 6: Functional assessment of CD8 T cells from blood and BAL samples in HIV infection. A. Gating strategy showing a representative plot of the live CD3+ and CD8+ T cells, and the proportion of these cells producing IFN-γ and IL-17A in either blood or BAL samples in the presence or absence of PMA/Ionomycin stimulation. **B.** Intracellular cytokine staining scatter plots with bars (median) and whiskers (IQR) showing the percentage of CD8+ T-cells that produce IFN-γ and IL-17A under no mitogen stimulation condition in samples from blood and BAL compartments in PLWH and those without HIV. **C.** Representative plot showing the gating strategy of a proportion of live CD3+ and CD8+ T cells expressing PD-1 (unstained FMO control, left panel) and PD-1+ (right panel). **D.** Scatter plots with bars (median) and whiskers (IQR) illustrating protein level expression of PD-1+ by CD8+ T-cells from blood and BAL samples in PLWH and those without HIV.
